# Feasibility of a Web-Based Survey of Hallucinations and Assessment of Visual Function in Patients With Parkinson’s Disease

**DOI:** 10.2196/ijmr.2744

**Published:** 2014-01-06

**Authors:** Mary Lou Jackson, Peter J Bex, James M Ellison, Paul Wicks, Jennifer Wallis

**Affiliations:** ^1^Massachusetts Eye and Ear InfirmaryDepartment of OphthalmologyHarvard Medical SchoolBoston, MAUnited States; ^2^Schepens Eye Research Institute, Massachusetts Eye and Ear InfirmaryDepartment of OphthalmologyHarvard Medical SchoolBoston, MAUnited States; ^3^McLean HospitalDepartment of PsychiatryHarvard Medical SchoolBoston, MAUnited States; ^4^PatientsLikeMe IncResearch and DevelopmentCambridge, MAUnited States

**Keywords:** Parkinson’s disease, hallucinations, contrast sensitivity, Charles Bonnet Syndrome

## Abstract

**Background:**

Patients with Parkinson’s disease (PD) experience visual hallucinations, which may be related to decreased contrast sensitivity (ie, the ability to discern shades of grey).

**Objective:**

The objective of this study was to investigate if an online research platform can be used to survey patients with Parkinson’s disease regarding visual hallucinations, and also be used to assess visual contrast perception.

**Methods:**

From the online patient community, PatientsLikeMe, 964 members were invited via email to participate in this study. Participants completed a modified version of the University of Miami Parkinson’s disease hallucinations questionnaire and an online vision test.

**Results:**

The study was completed by 27.9% (269/964) of those who were invited: 56.9% of this group had PD (153/269) and 43.1% (116/269) were non-Parkinson’s controls. Hallucinations were reported by 18.3% (28/153) of the Parkinson’s group. Although 10 subjects (9%) in the control group reported experiencing hallucinations, only 2 of them actually described formed hallucinations. Participants with Parkinson’s disease with a mean of 1.75 (SD 0.35) and the control group with a mean of 1.85 (SD 0.36) showed relatively good contrast perception as measured with the online letter test (*P*=.07). People who reported hallucinations showed contrast sensitivity levels that did not differ from levels shown by people without hallucinations (*P*=.96), although there was a trend towards lower contrast sensitivity in hallucinators.

**Conclusions:**

Although more Parkinson's responders reported visual hallucinations, a significant number of non-Parkinson's control group responders also reported visual hallucinations. The online survey method may have failed to distinguish between formed hallucinations, which are typical in Parkinson's disease, and non-formed hallucinations that have less diagnostic specificity. Multiple questions outlining the nature of the hallucinations are required. In a clinical interview, the specific nature of the hallucination would be further refined to rule out a vague description that does not indicate a true, formed visual hallucination. Contrary to previous literature, both groups showed relatively good contrast sensitivity, perhaps representing a ceiling effect or limitations of online testing conditions that are difficult to standardize. Steps can be taken in future trials to further standardize online visual function testing, to refine control group parameters and to take steps to rule out confounding variables such as comorbid disease that could be associated with hallucinations. Contacting subjects via an online health social network is a novel, cost-effective method of conducting vision research that allows large numbers of individuals to be contacted quickly, and refinement of questionnaires and visual function testing may allow more robust findings in future research.

## Introduction

Parkinson’s disease (PD) is a disorder that typically occurs in mid to late life, with symptoms including tremor, rigidity, bradykinesia, stooped posture and shuffling gait. Approximately 1/3 of PD patients report visual hallucinations such as seeing people or animals [1,2]; thought to be either related to the disease or pharmacological treatments [3].

Hallucinations are also experienced by patients with reduced vision, and this is known as Charles Bonnet Syndrome (CBS) [4-6]. CBS patients usually retain insight into the hallucinatory nature of their visual experiences [7] yet hesitate to discuss the symptom with health care providers for concern of being viewed as mentally ill [8]. A prospective evaluation of 224 patients presenting for vision rehabilitation identified a high prevalence of CBS (33%) and a correlation with impaired contrast sensitivity (CS), the ability to discern shades of grey [7,9]. Patients reported seeing formed images of different things such as animals, faces, patterns or other objects. PD patients who report visual hallucinations are reported to have poorer CS using vision tests in a controlled clinical environment [10]. It is arduous and costly to conduct large trials of patients to assess correlates of hallucinations and visual functions, and as a result, quicker novel methods of conducting research are desirable.

PatientsLikeMe (PLM) is an online platform with over 190,000 members which offers patients tools to track their illness, share their data with peers and participate in research studies. There is evidence suggesting that use of the platform may even benefit patient outcomes [11]. PLM has an advanced system for patients with PD that offers the ability to use a patient-reported version of the Unified Parkinson’s Disease Rating Scale (UPDRS-III) [12] to record the impact of their condition over time. Comparison of such data with clinical trial data suggests a high degree of week to week variability in PD symptoms reported on the platform [13]. Members of the PLM PD community have previously contributed to research in sensitive areas such as pathological gambling [14] using a built-in survey tool. Hallucination is also a sensitive topic for many patients who have insight into the unreal nature of what they see and, therefore, the online survey method was of interest.

Ongoing clinical evaluation and testing for patients with PD are resource intensive. Recent efforts to address this include telemedicine allowing “virtual house calls” for PD patients [15]. Enrolling and conducting the vision test with a large sample of PD patients can also be difficult and resource intensive [15]. In this study we aimed to explore the feasibility of using an online platform to examine the relationship between reported hallucinations and contrast perception in patients with Parkinson’s disease compared to controls.

## Methods

### Enrollment

This study used an online survey of visual hallucinations with a standardized questionnaire and a novel test of contrast sensitivity. After institutional review board approval by the Human Studies Committee at the Massachusetts Eye and Ear Infirmary, email invitations were sent to members of the PLM community. The protocol and the study complied fully with the declaration of Helsinki. Patients who were members of the PLM community had previously self-identified themselves as being diagnosed with Parkinson’s disease. If patients chose to participate in the study, they were asked to click on an email link to access the consent document, and after reading the consent, to click on a link to indicate their agreement to participate. If no response was received, participants were sent an automated reminder message after three days.

### Online Survey of Hallucinations

Participants completed the University of Miami Parkinson’s disease hallucinations questionnaire (UM-PDHQ), which had been previously developed by Papapetropoulos et al to assess and characterize hallucinations in Parkinson’s patients [16]. The questions enquired whether the individual experienced hallucinations, and asked questions about frequency, types, and insight into the unreal nature of the images. Three questions in the UM-PDHQ clarified the nature of the hallucinations, (ie, whether they were solid, colored, and normal in size). Three additional questions were added to the UM-PDHQ regarding history of eye disease and whether hallucinations were monocular or binocular (see Multimedia Appendix 1). Patients participating in this study were encouraged to discuss the symptom with their physician if they had concerns or questions.

### Contrast Sensitivity Test

**Figure 1:**
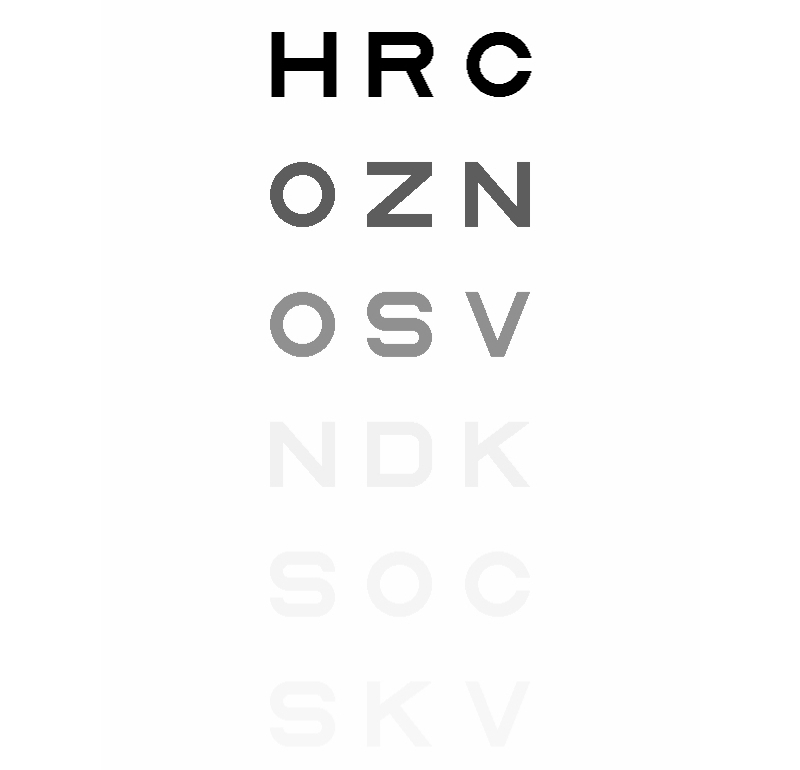
The contrast sensitivity test stimuli letters. A total of 16 rows of letter triplets were presented to the respondents, with row 1 letter triplet being of highest contrast and row 16 letter triplet being of lowest contrast. Represented from top to bottom are row 1, row 2, row 3, row 9, row 10 and row 11 of the letter triplets.

Patients completed a vision test of contrast sensitivity that was similar to a commonly used test, the Pelli-Robson chart [17]. Three letters of the same contrast were shown and the subject was asked to type the letters seen. Each subsequent triplet was of reduced contrast (Figure 1). If no letters could be detected, the participant would receive a score of 0.00, and the highest possible score was 2.25. A person with normal vision would score 1.95. The size of the letters created a 0.5° target viewed on a monitor with an average of 44 pixels/cm at a typical viewing distance of 56 cm [18]. The luminance of the display was assumed to have a gamma value of 2.0 (gamma=1.8 for Mac OS and gamma=2.2 for Windows OS) [19], with 8 bit grey scale resolution (256 luminance levels). Letters were presented in triplets of the same contrast and contrast decreased by a factor of 1/√2 each line. Regardless of the brightness setting of the subject’s display, maximum achievable Michelson contrast (L_min_=0, L_max_=255) approached 100% and minimum achievable Michelson contrast (L_min_=254, L_max_=255) approached 0.02%. The worst case would be a 6 bit display where the lowest presentable Michelson contrast would be 1.2%, (L_min_=63, L_max_=64). There are many assumptions in these values, and the contrast sensitivity assessment can only be approximate. Nevertheless, small changes in letter size, gamma and brightness have relatively limited effect on presented contrasts, composed of only 2 values (light grey letter on white background), and there is no reason to assume that there was any systematic difference in the displays used by different subject groups. Consequently, these data allowed a crude classification of contrast sensitivity.

### Statistical Analysis

The main comparison assessed the impact of PD and hallucinations on contrast sensitivity. Results from the UM-PDHQ divided participants into the following three goups: (1) currently hallucinating, (2) reported never having hallucinations, (3) and those who hallucinated previously but not within the past month. Preliminary analyses showed that the results did not change when collapsing the group “currently hallucinates” and “previously hallucinated”; thus, a two-way between-groups analysis of variance was conducted to explore the impact of Parkinson’s disease (PD vs no PD) and hallucinations (currently/previously vs never hallucinated) on contrast sensitivity. Chi-square tests of independence were performed to examine potential differences in proportions of PD versus control subjects. All analyses were computed using SPSS (version 17.0).

## Results

### Enrollment

Email invitations were sent to 964 PLM members: 482 with PD and 482 controls (Table 1). At the end of data collection (14 days after email invitation), 269 had completed the study: 153 PD subjects and 116 controls, including 80 patients with amyotrophic lateral sclerosis and 36 with depressive disorder.

### Contrast Sensitivity Test

Both groups, participants with PD (mean 1.75, SD 0.35) and the control group (mean 1.85, SD 0.36), showed relatively good CS, and there was no difference between the groups (main effect of Parkinson’s disease, *P*=.07). Neither the control nor the PD group showed a difference in CS between those who reported and did not report hallucinations (interaction Parkinson’s disease and hallucinations, *P*=.96).

### Data: Online Survey of Hallucinations

As seen in Table 1, 18.3% (28/153) of PD patients and 9% (10/116) of controls reported current visual hallucinations (*P*=.004). Hallucinations reported by participants with PD included mice, cats, people, distorted faces, furniture or complex patterns. Only 2 of the 10 control subjects reported formed images and the remainder reported seeing lights, vague peripheral images or hallucinations that were related to drug ingestion.

**Table 1:** Parkinson’s and control group characteristics (N=269).

		Controls n=116	Parkinson’s disease n=153
Age (years), mean (SD, range)		51.8 (10.2, 21-74)	61.5 (9.6, 36-86)
**Gender, n (%)** ^a^			
	Female	70 (60.3)	87 (56.9)
	Male	45 (38.8)	64 (41.8)
Contrast sensitivity (score), mean (SD)		1.85 (0.36)	1.75 (0.35)
Currently hallucinating, n (%)		10 (9)	28 (18.3)
Previously hallucinated, n (%)		16 (13.8)	23 (15.0)
Never hallucinated, n (%)		90 (77.6)	102 (66.7)

^a^Gender not reported by 1 control and 2 PD subjects.

## Discussion

### Principal Results

This research confirms the feasibility of conducting rapid online vision testing and efficiently gathering questionnaire data from large numbers of individuals; however, it also points to limitations of such research. Our findings confirm previous reports describing that hallucinations exist in PD patients [1] and the number of subjects reporting hallucinations is similar to previous clinical research suggesting that the online tool was successful for this patient group. However, the report of hallucinations by controls indicates that online questionnaires need to be more explicit to gain accurate reports in this group. Two of the authors (MLJ, JE) have extensive clinical experience interviewing patients with a symptom of hallucinations and such clinical experience suggests that targeted questioning is required to discern true visual hallucinations, illusions of mistaking items, or vague complaints that do not fulfill the criteria for formed hallucinations.

### Limitations

Control subjects’ descriptions of hallucinations were vague, or attributed to medications, in more than 80%, hence the rate of hallucinations in the control group was exaggerated. In-person interview would have clarified these reports. It is also possible that the study attracted those who were interested in the topic of hallucinations and this may have also contributed to over-reporting of such a symptom in control subjects. A limitation of this study was that the control group was “disease controls” rather than “healthy controls” as these were the most convenient comparison sample available from PLM at the time. This could be addressed in future trials by using a sample of caregivers who are members of the PLM community and, in addition, an attempt could be made to screen for both comorbid disease and cognitive status. No test of cognitive performance was used and PD subjects with less severe PD may have been selected. Hallucinations have been reported early in the PD disease course [3], but they may be less frequent in this group. As a result, this may have led to an underreporting of hallucinations in the PD group.

We did not find any statistically significant difference in contrast sensitivity between PD patients and controls. This is in contrast to previous literature that shows PD patients have reduced contrast sensitivity. Our study may have suffered from selection bias in that subjects with good contrast sensitivity may be those who continue to use a computer, while subjects with poor contrast sensitivity may not use a computer at all. In future trials the severity of PD could be ascertained. A further reason for our failure to dissociate the two groups might be that the test was too easy (ie, ceiling effect); however, it is worth noting that later triplets were very difficult to see even for the experimenters. The lack of standardization of computer monitors, home lighting, and the distance participants sit from their monitors are factors that require attention in future trials. More precise directions for vision testing may lead to greater standardization of the online testing. Novel methods of standardization for spatial frequency, for example, could include matching a common object such as dollar bill to a shape on the screen. Future research could compare online and in-person results to validate online methods.

### Potential Benefits

Collecting data from online patient communities offers potential to create new knowledge from the real-world experience of patients and; therefore, the techniques used in this study will become increasingly important as costs of research are increasingly scrutinized. These research methods may become very important in future years to reduce the burden of conducting research using in-person examinations, and to shorten the time of conducting trials. We have demonstrated a novel research that combines patient reported symptoms and measured visual function, and this method can be further expanded in the future to understand the symptom of hallucinations in patients who retain insight into the unreal nature of what they see.

### Conclusions

This research identified that hallucinations are more common in PD than controls but did not show a relationship between hallucinations and measured contrast perception. Our research directs how useful data may be collected in future studies as more standardized online techniques are developed. This research is an example of using an online community to conduct a survey of symptoms and vision that is quick, economical, and convenient for subjects. This has great potential for future online research. Patients are often hesitant to discuss hallucinations with a health care professional. In fact, we are certain that the majority of patients with CBS do not voluntarily report such symptoms to clinicians out of concern that they will be regarded as mentally ill [7,8]. Our pilot study shows the feasibility of enrolling and collecting data about hallucinations in this patient group. In addition, a survey such as the one used in this study offers patient education. Patients may not volunteer the symptom of hallucinations to their physicians due to the fear of being labeled as having a mental disorder. However, in our survey patients were advised that such a symptom may not indicate mental incompetence, hence reducing the stigma of reporting the symptom. Perhaps future research can determine if patients who participate in such a study do volunteer the symptom to physicians subsequently.

## Multimedia Appendix 1

A screenshot of the PatientsLikeMe online survey distributed to all participants.

## Abbreviations

CBS: Charles Bonnet Syndrome
CS: Contrast sensitivity
PD: Parkinson’s disease
PLM: PatientsLikeMe
UM-PDHQ: University of Miami Parkinson’s Disease Hallucinations Questionnaire
