# Supplementary material for: Feasibility of a Web-Based Survey of Hallucinations and Assessment of Visual Function in Patients With Parkinson’s Disease
Source: Interact J Med Res. 2014 Jan 6;3(1):e1. doi: 10.2196/ijmr.2744 (PMC3906694; doi:10.2196/ijmr.2744)
Supplement: Supplementary file 1 [file ijmr_v2i2e30_app1.pdf]

PatientsLikeMe Survey Outline
How is your vision? (v4c)

Dear PatientsLikeMe member,

PatientsLikeMe is collaborating with researchers at Massachussetts Eye & Ear Infirmary to conduct a new research survey. You are being asked to participate in a research study about your experience of visual hallucinations and your ability to see items when they do not have high contrast.

You may know that some patients with certain disorders can experience visual hallucinations (seeing things when nothing is there), or seeing 'phantom' visions. They may see patterns, shapes or vivid images of objects. The purpose of this study is to better understand how these 'phantom visions' are related to vision. You will be asked to complete a 10-minute survey that includes questions about your experience of hallucinations, whether or not you yourself have experienced them.

You also will be asked to do simple tests of your ability to see black and white. Some of the targets are more black and others more grey. The results from the survey will be shared with researchers at the Massachusetts Eye and Ear Infirmary Vision Rehabilitation Clinic. The survey results which can be linked to you personally will be kept confidential, according to the PLM Privacy Policy. Individual survey results that are sent to Mass Eye and Ear will not contain any information that could identify you. The results may be presented in educational settings, at professional conferences, or published in professional journals in the field of medicine. No personal information will be presented in any way and all individual results will be kept confidential.

No risks or discomforts are anticipated from taking part in this study. It is voluntary to participate in this survey. You can withdraw from the study at any time. If you decide not to participate in the survey or if you decide to not complete the survey after you start it, you will still remain a member in good-standing of the PatientsLikeMe community. If you request, we will send you a summary of the project findings.This summary will be of overall findings from all of the participants.

If you have questions or concerns about this study, please contact PatientsLikeMe at research@patientslikeme.com. The ethical aspects of this research project have been approved by the Human Studies Committee of the Massachusetts Eye and Ear Infirmary.

If you have any questions about your rights as a research subject or if you have questions, concerns or complaints about the research, you may contact: The Office of Research Administration 243 Charles Street Boston, MA, 02114 Telephone: 617 573 3446

If you agree to participate please click the link below.

Sincerely,

Paul Wicks, PhD

R&D Director

PatientsLikeMe

Section 1. **Hallucination screener**

1. **Do you experience any visual hallucinations (seeing things that are not really there or that other people do not see)?**

   Key: VISUALHALLUCINATIONS
   Format: Multiple-choice

   - No, I have never had visual hallucinations (Value: 0)
   - Yes (Value: 1)
   - Not within the past month, but it has happened in the past (Value: 2)

2. **Do you experience any other hallucinations (hear, smell, or taste things that are not really there or that other people do not experience)?**

   Key: NONVISHALSCREENER
   Format: Multiple-choice

   - Yes (Value: 1)
   - No (Value: 0)

Section 2. **Visual Hallucinations detail (Q2-Q6)**

Show if: (#VISUALHALLUCINATIONS IS '1') OR (#VISUALHALLUCINATIONS IS '2')

1. **How often do / did you experience visual hallucinations?**

   Key: HALFREQ
   Format: Multiple-choice

   - Experienced it only a few times (Value: 1)
   - Occasionally (less than once a week) (Value: 2)
   - Often (about once per week) (Value: 3)
   - Frequently (several times per week but less than once per day) (Value: 4)
   - Very frequently (once per day or more) (Value: 5)

2. **On average, how long do / did these visual hallucinations last?**

   Key: HALDURATION
   Format: Multiple-choice

   - Short duration (less than one second) (Value: 1)
   - Medium duration (less than ten seconds) (Value: 2)
   - Prolonged duration (more than ten seconds) (Value: 3)

3. **Do / did you think what you are seeing is real?**

   Key: HALREAL
   Format: Multiple-choice

   - Not real (Value: 0)
   - Sometimes real (Value: 1)
   - Always real (Value: 2)

4. **How many types of images do / did you experience?**

   Key: HALNUMIMAGES
   Format: Multiple-choice

   - One (Value: 1)
   - A few (2 or 3) (Value: 2)
   - Several (more than 3) (Value: 3)

5. **How severe/emotionally distressing do / did you find these images or visions?**

   Key: HALDISTRESS
   Format: Multiple-choice

   - No effect / friendly (Value: 1)
   - Mildly - produce little distress (Value: 2)
   - Moderately - produce distress and are disturbing and disruptive (Value: 3)
   - Severely - very disturbing (medications may be required) (Value: 4)

Section 3. **Q7 Eye disease**

1. **Have you been diagnosed with any eye disease? (i.e. near or far sight problems, double vision, cataract, macular degeneration, glaucoma, retinitis, retinal detachment, diabetic or hypertensive eye disease)**

   Key: EYEDISEASE
   Format: Multiple-choice

   - Yes (Value: 1)
   - No (Value: 0)

Section 4. **Q7 Describe eye disease**

Show if: (#EYEDISEASE IS '1')

1. **In the previous section, you said that you had been diagnosed with an eye condition. Please describe this in the space below.**

   Key: OPENTEXTEYEDISEASE
   Format: Free-form text

Section 5. **Q8 Current medications**

Show if: (#VISUALHALLUCINATIONS IS '1') OR (#VISUALHALLUCINATIONS IS '2')

1. **What are your current medications?**

   Key: OPENTEXTCURRENTMEDS
   Format: Free-form text

   Please list below

Section 6. **Q9 Recent Change in Medications**

Show if: (#VISUALHALLUCINATIONS IS '1') OR (#VISUALHALLUCINATIONS IS '2')

1. **Was there a recent change in your medications?**

   Key: MEDSCHANGE
   Format: Multiple-choice

   - Yes (please describe below) (Value: 1)
   - No (Value: 0)

2. **If yes, what was the change in your medication? Otherwise write "no change"**

   Key: OPENTEXTCHANGEMEDS
   Format: Free-form text

Section 7. **Q10 - Reason for recent change in meds**

Show if: (#MEDSCHANGE IS '1')

1. **In the previous section you said you had recently changed your medication. Was this change related to the appearance or change in the characteristics of your hallucinations?**

   Key: REASONCHANGEMEDS
   Format: Multiple-choice

   - Yes (Value: 1)
   - No (Value: 0)
   - I cannot tell (Value: 3)
   - Not applicable (Value: 4)

Section 8. **Q11 - On/Off fluctuations**

Show if: (#VISUALHALLUCINATIONS IS '1') OR (#VISUALHALLUCINATIONS IS '2')

1. **Do you experience hallucinations while "on" or "off" certain medications?**

   Key: HALONOFFMEDS
   Format: Multiple-choice

   - When I am on medications (Value: 1)
   - When I am off medications (Value: 2)
   - They can happen anytime - not related to being on or off medications (Value: 3)

Section 9. **Q12 - Hallucinations detail**

Show if: (#VISUALHALLUCINATIONS IS '1') OR (#VISUALHALLUCINATIONS IS '2')

1. **What do your hallucinations look like?**

Key: HALLOOKLIKE
Format: Check all that apply

Check all that apply

- Not formed / cannot describe (Value: 0)
- Whole faces (Value: 1)
- Fragmented faces (Value: 2)
- Whole people (Value: 3)
- Animals, insects, reptiles (Value: 4)
- Objects (Value: 5)
- Other (please describe below) (Value: 6)

2. **If you see hallucinations of faces or people, are they familiar (people known to you) or unfamiliar (people that you do not know)?**

   Key: HALFACES
   Format: Check all that apply

   Check all that apply

   - I don't hallucinate faces or people (Value: 0)
   - They are familiar people known to me (Value: 1)
   - They are unfamiliar people who I do not know (Value: 2)

3. **Please describe your visual hallucinations in the box below**

   Key: OPENTEXTDESCRIBEHAL
   Format: Free-form text

Section 10. **Q13-16 Hallucination quality details**

Show if: (#VISUALHALLUCINATIONS IS '1') OR (#VISUALHALLUCINATIONS IS '2')

1. **Is there anything you can do to make the images from your hallucinations disappear?**

   Key: HALDISAPPEAR
   Format: Multiple-choice

   - Yes (please describe below) (Value: 1)
   - No (Value: 2)

2. **If you answered "yes" to the question above, please describe below what you can do to make the visual hallucinations disappear. Otherwise, leave this box blank.**

   Key: OPENTEXTHALDISAPPEAR
   Format: Free-form text

3. **Are hallucinations seen in one eye only, or with both eyes?**

   Key: HALONEBOTHEYES
   Format: Multiple-choice

   - One eye only (left) (Value: 1)
   - One eye only (right) (Value: 2)
   - Both eyes (Value: 3)
   - I'm not sure (Value: 4)

4. **Is there any particular time of day or particular lighting conditions when you are more likely to experience visual hallucinations?**

   Key: HALTIMEOFDAY
   Format: Check all that apply

   Check all that apply

   - No particular time or situation (Value: 0)
   - During the day, or when it's bright (Value: 1)

- During the night, or when it's dark (Value: 2)
- Anytime when the light is dim (Value: 3)

5. **Do the images ever make any sound or noise?**

   Key: HALAUDITORY
   Format: Multiple-choice

   - Yes (Value: 1)
   - No (Value: 0)

6. **Do the images ever move?**

   Key: HALMOVE
   Format: Multiple-choice

   - Yes (Value: 1)
   - No (Value: 0)

7. **Do you experience the hallucinations:**

   Key: HALCONSCIOUSNESS
   Format: Check all that apply

   (Check all that apply)

   - when you are wide awake (Value: 1)
   - when you are drowsy (Value: 2)
   - when you wake up during the night (Value: 3)
   - at any time of day (Value: 4)

Section 11. **Q17-20 Hallucination quality details**

Show if: (#VISUALHALLUCINATIONS IS '1') OR (#VISUALHALLUCINATIONS IS '2')

1. **Are the images normal size?**

   Key: HALSIZE
   Format: Check all that apply

   Check all that apply

   - Yes (Value: 0)
   - No, they are smaller than normal (Value: 1)
   - No, they are larger than normal (Value: 2)

2. **Are the images transparent or solid?**

   Key: HALTRANSPARENT
   Format: Check all that apply

   Check all that apply

   - Transparent (Value: 1)
   - Solid (Value: 2)

3. **Are the images colored?**

   Key: HALCOLORS
   Format: Check all that apply

   Check all that apply

   - Yes, they are colored (Value: 1)
   - No, they are black and white (Value: 2)

4. **Is the onset of visual hallucinations gradual or sudden?**

Key: HALONSET
Format: Check all that apply

Check all that apply

- Gradual (they appear and disappear slowly) (Value: 1)
- Sudden (they appear and disappear suddenly) (Value: 2)
- I cannot tell (Value: 3)

Section 12. **Q21 - 23 All respondents**

1. **Do you have more difficulty than others reading pale print, such as the newspaper, or seeing colored print?**

   Key: PALEPRINT
   Format: Multiple-choice

   - Yes, a great deal more difficulty (Value: 1)
   - Yes, some more difficulty than others (Value: 2)
   - No (Value: 0)

2. **Do you have more difficulty than others seeing steps or curbs?**

   Key: STEPSCURBS
   Format: Multiple-choice

   - Yes, a great deal more difficulty (Value: 1)
   - Yes, some more difficulty than others (Value: 2)
   - No (Value: 0)

3. **Do you find that you need more lighting than others?**

   Key: LIGHTING
   Format: Multiple-choice

   - Yes, much more than others (Value: 1)
   - Yes, a little more than others (Value: 2)
   - No, no more than others (Value: 0)

Section 13. **Contrast sensitivity 1**

In this part of the study, we will be showing you a series of 3-letter rows of text. For each row, please type as many letters as you can see. If you can't see a letter clearly, please make your best guess.

Please sit at arm's length from your computer screen. Make sure you are directly in front of the screen

1. **Row 1 - Please type all the letters that you can see in the text box below.**

   Key: NCD1
   Format: Free-form text

   <p></p>

2. **Row 2 - Please type all the letters that you can see in the text box below.**

   Key: HON2
   Format: Free-form text

   <p></p>

3. **Row 3 - Please type all the letters that you can see in the text box below.**

Key: HNV3
Format: Free-form text

<p></p>

4. **Row 4 - Please type all the letters that you can see in the text box below.**

   Key: SZR4
   Format: Free-form text

   <p></p>

5. **Row 5 - Please type all the letters that you can see in the text box below.**

   Key: CVN5
   Format: Free-form text

   <p></p>

6. **Row 6 - Please type all the letters that you can see in the text box below.**

   Key: OHS6
   Format: Free-form text

   <p></p>

7. **Row 7 - Please type all the letters that you can see in the text box below.**

   Key: ZCK7
   Format: Free-form text

   <p></p>

8. **Row 8 - Please type all the letters that you can see in the text box below.**

   Key: OVZ8
   Format: Free-form text

   <p></p>

Section 14. **Contrast sensitivity 2**

In this part of the study, we will be showing you a series of 3-letter rows of text. For each row, please type as many letters as you can see. If you can't see a letter clearly, please make your best guess.

Please sit at arm's length from your computer screen. Make sure you are directly in front of the screen

1. **Row 9 - Please type all the letters that you can see in the text box below.**

Key: SVD9
Format: Free-form text

<p></p>

2. **Row 10 - Please type all the letters that you can see in the text box below.**

   Key: VSH10
   Format: Free-form text

   <p></p>

3. **Row 11 - Please type all the letters that you can see in the text box below.**

   Key: HNK11
   Format: Free-form text

   <p></p>

4. **Row 12 - Please type all the letters that you can see in the text box below.**

   Key: ZNR12
   Format: Free-form text

   <p></p>

5. **Row 13 - Please type all the letters that you can see in the text box below.**

   Key: SDC13
   Format: Free-form text

   <p></p>

6. **Row 14 - Please type all the letters that you can see in the text box below.**

   Key: 14
   Format: Free-form text

   <p></p>

7. **Row 15 - Please type all the letters that you can see in the text box below.**

   Key: 15
   Format: Free-form text

   <p></p>

8. **Row 16 - Please type all the letters that you can see in the text box below.**

   Key: 16
   Format: Free-form text

   <p></p>

Section 15. **Condition**

1. **Please list any medical conditions that you have been diagnosed with**

   Key: *none*
   Format: Free-form text

Section 16. **Finale**
The survey is nearly complete. Thank you for taking part. We will share the results with you shortly when we have analyzed them. To complete the study, please hit "submit survey" at the bottom of the page.

1. **If you have any comments or questions for us about this survey, please enter them here, or else hit "submit survey" to complete the study. Thank you again!**

   Key: FINALE
   Format: Free-form text

Generated Aug 21, 2012 05:08AM

© 2005-2012 PatientsLikeMe. All Rights Reserved. Information on PatientsLikeMe.com does not constitute medical advice.
Information found on PatientsLikeMe is based on reports from PatientsLikeMe members.
